# Changes of flowering phenology and flower size in rosaceous plants from a biodiversity hotspot in the past century

**DOI:** 10.1038/srep28302

**Published:** 2016-06-17

**Authors:** Qin Yu, Dong-Rui Jia, Bin Tian, Yong-Ping Yang, Yuan-Wen Duan

**Affiliations:** 1 Key Laboratory for Plant Diversity and Biogeography of East Asia, Kunming Institute of Botany, Chinese Academy of Sciences, Kunming 650204, P. R. China; 2 China Plant Germplasm and Genomics Center, Kunming Institute of Botany, Chinese Academy of Sciences, Kunming 650201, P. R. China; 3 Institute of Tibetan Plateau Research at Kunming, Kunming Institute of Botany, Chinese Academy of Sciences, Kunming 650204, P. R. China; 4 University of the Chinese Academy of Sciences, Beijing 100049, P. R. China; 5 School of Ecology and Environmental Science, Yunnan University, Kunming 650091, P. R. China

## Abstract

Responses of plant traits to climate changes are complex, which could be mirrored by the investigations of herbarium specimens. By examining specimens of *Rosa* and *Cotoneaster* species collected since 1920s in Hengduan Mountains, we analyzed the changes of flowering phenology and flower size in the past century when climate changes were considered to be intensified. We found that flowering phenology of *Rosa* showed no significant change, but flowering phenology of *Cotoneaster* was delayed in recent years. Flower size of *Rosa* species showed a marginally significant decrease over the past century. The results suggested that responses of flowering time to global changes and pollinator mediated selection on floral traits might be more complex than what were expected. Our results indicated that future researches based on investigations of herbarium specimens should be carried out on multiple plant species with different flower structures and life histories to better understand the effects of climate changes on plant traits.

The global changes in the past century, mainly consisting of climate changes and intensified human activities, have been considered to be threatening biodiversity. Pollinators are a key component of global biodiversity and represent a key service for terrestrial ecosystems, which is vital to the maintenance of both wild plant communities^1^ and agricultural productivity^2^. There are compelling evidences of declines in pollinator richness and density at a global scale^3^, which has been attracting public and scientific concerns greatly since parallel declines were found in pollinators and insect-pollinated plant species^4^. However, it still remains unclear whether or not pollinator declines have driven the evolution of floral traits. Historical collections and/or records of plants and insects were useful to examine the population demographic changes in the time scale, such as plant traits^5^, plant flowering phenology^6^, pollinator richness and abundance^7^ and pollinator-plants interactions^4^,^8^,^9^. Therefore, examinations on flower size based on historical collections might reflect the selection by pollinators decline to a certain degree.

The Hengduan Mountains (HMs) in the Eastern Himalaya are considered to be the core region of the Himalaya hotspot, one of the global biodiversity hotspots^10^. The HMs, extending from north-west Yunnan, through west Sichuan and east Tibet, to south Qinghai, are renowned for their examples of phyletic radiations and concomitant high levels of endemic species and generic richness^11^. From 1920s to 2010s, the extensive and continuous collections of herbaria in this region by Chinese botanists provided an ideal opportunity to examine the changes of flowering phenology and flower size, which could jointly mirror the plant-pollinator relationships in the HMs in the past century. Accordingly, based on the investigations on herbaria of *Rosa* and *Cotoneaster* (Rosaceae) (Table 1), which were widely distributed in south part of the HM in northwest Yunnan province, we examined the changes of flowering phenology and flower size from 1920s to 2010s, with the aim of evaluating the effects of global changes on plants in the HMs in the past century.

## Results

We found that flowering phenology of *Rosa* was delayed significantly with the increase of altitude, but showed no significant change in the past century (Table 2). In addition, flowering phenology of *Cotoneaster* was delayed with the increase of altitude and in recent years (Table 2). Flower size of *Rosa* species, indicated by the petal length, showed a marginally significant decrease over the past century, but no significant change was found in the flower size with the increase of altitude (*P* = 0.06; Table 2).

## Discussion

Parallel to the global trend^12^, recent studies strongly suggested that the temperature increased significantly in the past century in the HMs^13^,^14^. Warming was considered to affect flowering phenology significantly, and there is a general trend of advanced phenology for both plants and animals^15^,^16^,^17^. However, in fact, experiments strongly suggested that the responses of plants were complex and species-dependent^18^,^19^,^20^. Although the flowering phenologies of *Rosa* and *Cotoneaster* species were delayed with the increase of altitude, indicating that temperature might still be one of the main factors affecting flowering time, they showed different changes in the past century. Specifically, flowering phenology of *Cotoneaster* species was delayed significantly in the recent years, but flowering phenology of *Rosa* species showed no significant changes. A recent study based on *Rhododendron* species from the HMs suggested that the flowering phenology showed no directional change over the past 45 years of rapid warming in this region, but responded significantly to the annual temperature deviation and the temperature deviation of the fall in the last years^6^. Therefore, our results emphasized the species dependent responses to warming in phonological events of plants, especially for the temperate trees whose flowering times are not primarily controlled by temperature^21^.

Pollination is an essential process in the sexual reproduction of seed plants, and about 88% of angiosperm species^22^ and 70% of crop species^2^ rely on animal pollination. Globally, although the drivers of pollinator loss were complicated^23^, it was widely accepted that pollinator species and abundance declined significantly in the past century^2^,^3^,^4^,^7^,^8^, which might result in the increased investment in plants to enhance attraction to pollinators. Large floral display size, consisting of flower size and number, was considered to be one of the most common strategy by plants to attract pollinators from a long distance^24^,^25^. However, flower size of *Rosa* species showed a decreasing trend in the past century, indicating a reduced investment on flower size in *Rosa*. This does not suggest a reduced attraction to pollinators because there are many flowers in blooming in *Rosa* species, which could contribute to the attractions to pollinators from a long distance. Accordingly, the reduced flower size of *Rosa* species might result from the reduced resource investments to flowers instead of selective pressures from the pollinators, but this speculation needs to be demonstrated in future researches.

Collectively, we suggested that responses of flowering time to global changes might be more complex than what were expected, and thus, under the backgrounds of global changes and pollinator decline^3^ and intensified pollinator competition in biodiversity hotspots^26^, pollinator mediated selection on floral traits that could be influenced by climate changes^27^, should also be complex. Conclusively, to clarify the changes of flowering phenology and flower size in time scale in the past century, future researches based on investigations of herbaria should be carried out on multiple plant species with different flower structures, numbers and life histories, given that plant species might be experiencing various and different selective pressures.

## Methods

We only located collections of *Rosa* and *Cotoneaster* that were preserved in herbaria of Kunming Institute of Botany (KUN) and occurred in northwest Yunnan province, including Nujiang, Dali, Lijiang and Diqing. Four criteria were applied to screen the herbaria: 1) the species should be collected in recent 10 years to ensure that we could get the recent data; 2) information, e.g. date of collection, altitude and phase (flowering and/or fruiting), should be specified; 3) only one herbarium should be employed for duplicate collections, which were made at the same time and same place; 4) the number of herbarium of each specie should be no less than 10. Four *Rosa* species and 7 *Cotoneaster* species were used for further analysis (Table 1). In total, 105 specimens of *Cotoneaster* species and 125 specimens of *Rosa* species were used to analyze flowering phenology, and 77 specimens of *Rosa* species were used for measurements of flower size.

As suggested by a previous study^6^, to combine the analysis across species, flowering time (day) and altitude (meters above sea level) of each collection were converted to within-species deviation from the mean. Specifically, flowering time was converted to relative flowering phenology by calculating days after (+) or before (−) mean collection date of each species, and altitude was also converted to relative altitude by calculating meters above (+) or below (−) the species mean altitude.

Flower size was measured on *Rosa* species only because flowers of *Cotoneaster* species on specimens were too small to be measured correctly. Both length and width of one petal of a given flower that was kept finely were measured using a digital caliper. Also, to combine the analysis across species, we converted flowers size to the relative flower size by dividing the data on each herbarium specimens by the mean of each species.

To analyze the trends of plant traits with altitude in the past century, we performed a linear regression with plant traits, with relative flowering phenology and relative flower size as dependent variables and relative altitude and year as independent variables. Furthermore, a significant positive relationship between petal length and width were observed (R = 0.61, *P* < 0.01), so we used petal length as an indicator of flower size.

## Additional Information

**How to cite this article**: Yu, Q. *et al*. Changes of flowering phenology and flower size in rosaceous plants from a biodiversity hotspot in the past century. *Sci. Rep.* **6**, 28302; doi: 10.1038/srep28302 (2016).

## Figures and Tables

**Table 1:** *Rosa* and *Cotoneaster* species for flowering phenology and flower size and their altitudinal range.

Species	Lowest altitude	Highest altitude	Aims
*R. longicuspis*	1600	3100	Flower phenology, flower size
*R. omeiensis*	2200	4060	Flower phenology, flower size
*R. sericea*	2350	3500	Flower phenology, flower size
*R. soulieana*	2300	3500	Flower phenology, flower size
*C. buxifolius*	1900	2800	Flowering phenology
*C. dielsianus*	1690	3400	Flowering phenology
*C. franchetii*	1560	3300	Flowering phenology
*C. microphyllus*	1880	4400	Flowering phenology
*C. pannosus*	1870	2700	Flowering phenology
*C. poluninii*	1200	2700	Flowering phenology
*C. subadpressus*	3100	3700	Flowering phenology
*C. turbinatus*	1650	2700	Flowering phenology

**Table 2:** Regression analysis of flowering phenology of *Rosa* and *Cotoneaster*, and flower size with altitude and year as explainable variables.

Traits	Standard coefficient		*R*	F	*P*
	Altitude	Year			
Flowering phenology of *Rosa*	**0.22 (0.02)**	−0.02 (0.80)	0.21	2.88	0.06
Flowering phenology of *Cotoneaster*	**0.33 (<0.01)**	**0.28 (<0.01)**	0.41	10.56	<0.01
Flower size of *Rosa*	0.14 (0.21)	−0.22 (0.06)	0.25	2.44	0.09

Standard coefficients were shown with P values in the brackets, and significant relationships were labelled in bold.
